# Markers of Futile Resuscitation in Traumatic Hemorrhage: A Review of the Evidence and a Proposal for Futility Time-Outs during Massive Transfusion

**DOI:** 10.3390/jcm13164684

**Published:** 2024-08-09

**Authors:** Mark M. Walsh, Mark D. Fox, Ernest E. Moore, Jeffrey L. Johnson, Connor M. Bunch, Joseph B. Miller, Ileana Lopez-Plaza, Rachel L. Brancamp, Dan A. Waxman, Scott G. Thomas, Daniel H. Fulkerson, Emmanuel J. Thomas, Hassaan A. Khan, Sufyan K. Zackariya, Mahmoud D. Al-Fadhl, Saniya K. Zackariya, Samuel J. Thomas, Michael W. Aboukhaled, the Futile Indicators for Stopping Transfusion in Trauma (FISTT) Collaborative Group

**Affiliations:** Futile Indicators for Stopping Transfusion in Trauma (FISTT) Collaborative Group, Indiana University School of Medicine—South Bend, South Bend, IN 46617, USA; markfox@iu.edu (M.D.F.); ernest.moore@dhha.org (E.E.M.); jjohns52@hfhs.org (J.L.J.); cbunch1@hfhs.org (C.M.B.); jmiller6@hfhs.org (J.B.M.); iplaza@hfhs.org (I.L.-P.); rbranca2@hfhs.org (R.L.B.); dwaxman@versiti.org (D.A.W.); sthomas@beaconhealthsystem.org (S.G.T.); dhfulkerson@beaconhealthsystem.org (D.H.F.); emmanueljthomas4@gmail.com (E.J.T.); hassaankhan149@gmail.com (H.A.K.); szackari@iu.edu (S.K.Z.); malfadhl@iu.edu (M.D.A.-F.); szackariya15@gmail.com (S.K.Z.); samueljthomas1@gmail.com (S.J.T.); maboukha@iu.edu (M.W.A.)

**Keywords:** futility, futile resuscitation, massive transfusion, 24 h mortality, trauma, algorithm, biomarkers, fibrinolysis

## Abstract

The reduction in the blood supply following the 2019 coronavirus pandemic has been exacerbated by the increased use of balanced resuscitation with blood components including whole blood in urban trauma centers. This reduction of the blood supply has diminished the ability of blood banks to maintain a constant supply to meet the demands associated with periodic surges of urban trauma resuscitation. This scarcity has highlighted the need for increased vigilance through blood product stewardship, particularly among severely bleeding trauma patients (SBTPs). This stewardship can be enhanced by the identification of reliable clinical and laboratory parameters which accurately indicate when massive transfusion is futile. Consequently, there has been a recent attempt to develop scoring systems in the prehospital and emergency department settings which include clinical, laboratory, and physiologic parameters and blood products per hour transfused as predictors of futile resuscitation. Defining futility in SBTPs, however, remains unclear, and there is only nascent literature which defines those criteria which reliably predict futility in SBTPs. The purpose of this review is to provide a focused examination of the literature in order to define reliable parameters of futility in SBTPs. The knowledge of these reliable parameters of futility may help define a foundation for drawing conclusions which will provide a clear roadmap for traumatologists when confronted with SBTPs who are candidates for the declaration of futility. Therefore, we systematically reviewed the literature regarding the definition of futile resuscitation for patients with trauma-induced hemorrhagic shock, and we propose a concise roadmap for clinicians to help them use well-defined clinical, laboratory, and viscoelastic parameters which can define futility.

## 1. Introduction

### 1.1. Blood Product Shortages Heighten the Need for Reliable Markers of Futile Resuscitation of Severely Injured Patients

“Are they bleeding because they are dying, or dying because they are bleeding?” [1].

Before presenting a systematic review of the literature regarding the definition of futile resuscitation (FR) for patients with trauma-induced hemorrhagic shock, it is necessary to describe the background upon which this attempt to define, with well-defined metrics, what constitutes futility in the care of trauma patients.

Defining futility in the acute setting for a severely bleeding trauma patient (SBTP) requires a brief review of the definition of futility as determined by historically used parameters.

The resuscitation of a patient in hemorrhagic shock who requires massive transfusion (MT) and is not responding can be compared to pouring water into a leaky bucket. This analogy derives from the Latin word for futile which is “futilis” [2]. Defining futility is a very difficult endeavor, which is dependent not only on the number of blood products administered per hour but also on the “complex integration of patient- and time-specific data”. In the setting of the hectic moments of resuscitation where the measurement of the likelihood of death is dependent on many variables, it is often very difficult to find consensus among practicing clinicians [3]. As a result, there is a need to provide practical recommendations which will allow for the declaration of futility in this group of patients [2]. Recent definitions of futility in the transfer of trauma patients include a conservative definition for futile transfers as patients who died, had comfort measures implemented, or were discharged to hospice within 48 h of transfer based on previous studies of different surgical populations [4,5]. For the purposes of this review, we refer to futility as death within 24 h of admission because most deaths of SBTPs occur in the first 24 h [6]. 

The coronavirus disease 2019 (COVID-19) pandemic reduced the supply of available blood products due to the interruption of an already dwindling donor pool [7,8,9,10,11,12,13]. Even prior to the COVID-19 pandemic, the increasing cost of procuring and processing blood products limited blood product acquisition [14]. Patients with severe injuries often demand significant quantities of blood products, thus presenting an issue of equitable resource distribution by potentially limiting these same therapeutic resources for other patients. The importance of limiting the overuse of blood products is especially apparent when considering the higher incidence of penetrating injury in the United States compared to Europe, as penetrating injuries consume a large percentage of emergency blood products in US urban trauma centers [10,15,16,17,18,19,20,21,22,23,24,25,26,27,28,29]. Thus, the recent scarcity has increased the need for blood product stewardship, particularly among SBTPs who require MT [7,9,10,13,30,31,32]. 

The United States’ blood shortage has been further exacerbated by the empiric administration of fixed ratios of packed red blood cells (PRBCs), fresh frozen plasma (FFP), and platelets (PLTs). The fixed ratio strategy, combined with the recent adoption of whole blood (WB) by many urban trauma centers, has been associated with intermittent and unpredictable local shortages during peak moments of MTs [9,24,32,33,34,35,36,37]. The modern resuscitation of exsanguinating trauma patients applies a 1:1:1 or 1:1:2 ratio of FFP/PLTs/PRBCs [9,37,38,39]. This strategy of rapidly providing large quantities of blood products has resulted in studies attempting to identify reliable laboratory and clinical parameters which indicate when MT is no longer beneficial and thus futile [13,30,31,40,41].

Hospitals often transfuse more blood products than necessary, and the lack of clear and consistent clinical thresholds accentuates blood product overutilization [14]. When deciding whether to end a resuscitation attempt for suspected futility, traumatologists often face an ethical decision based on clinical gestalt in a time-sensitive and hectic environment of rapid administration of large quantities of blood products [9,18,42,43]. The recently heightened search for immediately available bedside markers of futility has led traumatologists to revisit the concept of transfusion “cut-points” as reliable markers of FR in SBTPs. Yet, it has become clear that the quantity of blood alone is an inadequate metric to determine futility without complementary clinical and laboratory measurements [9,24,30,31,32,40,41,44,45,46,47,48,49,50,51,52,53]. 

### 1.2. The Search for Reliable Markers of Futility Is Confounded by Varying Transfusion Practices and Measurements 

Although the literature on the early identification of FR for bleeding trauma patients has increased in recent years, the data cannot be easily compared among studies. Specifically, the identification of quantitative transfusion cut-points beyond which resuscitation will not be beneficial is complicated by the varied definitions of a unit of blood. Blood products per hour historically only counted the units of PRBCs. Recently, there has been an emphasis on identifying not only the number of units of PRBCs per hour, but also the number of units of FFP, PLTs, cryoprecipitate, and factor concentrates. The literature is further confounded by the significant increase in prehospital and emergency department (ED) use of WB with blood components. An additional discrepancy lies in how much blood product is in a single unit of WB, cryoprecipitate, FFP, and PLTs. The heterogeneity of defining units per hour coupled with differing local MT practices when applied to the multifaceted nature of trauma are a few major reasons why the definition of futility markers is not easily stratified into larger datasets amenable to meta-analysis [7,9,10,13,24,31,32,36,41,49,54,55,56,57].

Many scoring systems are applied in prehospital combat settings which include physiologic parameters and blood products per hour transfused. The optimal parameters in these scoring criteria remain ambiguous in the military combat aid station setting, which reflects the same ambiguities in the civilian ED or inpatient cohorts [58]. Defining futility in the civilian population remains unclear. However, the nascent Suspension of Transfusion and Other Procedures (STOP) criteria was recently derived from a civilian cohort [41]. The STOP criteria use a combination of return of spontaneous circulation (ROSC), the Glasgow Coma Scale (GCS), arrival systolic blood pressure (SBP), thromboelastography lysis at 30 min (TEG LY30), and lactate levels in attempt to predict futility.

The search for markers immediately available at the bedside has focused on quantitative transfusion cut-points, clinical markers, e.g., age, SBP, GCS, and laboratory markers (e.g., lactate, pH, and base deficit). However, until recently with the STOP criteria, no set of markers has reliably predicted FR for bleeding trauma patients. A seemingly obvious marker of FR is the number of blood products transfused per hour, but it has failed to accurately predict futility in retrospective studies. Data from older MT studies associate high mortality rates with transfusions of more than 50 U of blood components, whereas recent studies report increased survival rates, complicating the analysis of FR cut-points and leading to greater amounts of blood products being consumed in often futile situations [9,30,31,32,36,41,53,59,60].

The accuracy of determining markers for the initiation of MT does not carry the same weight as the decision to stop resuscitation since cessation of MT due to anticipated futility results in certain death for the patient. Therefore, compared to the threshold for initiating MT, the threshold for determining futility mandates a higher degree of accuracy and precision. Predicting FR in non-traumatic cardiopulmonary arrest has well-defined criteria unlike patients that present with traumatic injury. The goal would be to produce a set of markers with a 100% positive predictive value (PPV) so that the presence of these markers for futility only captures patients who would certainly die regardless of continued resuscitation, as well as a set of markers with 100% specificity, meaning that there would be no false positives for declaring the patients’ resuscitation futile, when in fact, they would live with continued resuscitation. With the understanding that clinical markers will not allow for the total removal of decisions based on clinical gestalt, it is our hope to provide a review of the recently proposed and possibly more accurate markers to better guide clinicians to appropriate cessation of resuscitation efforts [18,30,31,40,41,61].

Despite the widespread use of MT and UMT in these critically bleeding trauma patients, the mortality rates remain between 40 and 80%. Because of the limited availability of blood, which is commonly transfused in futile cases, recent studies have attempted to define transfusion cut-points for these patients. In spite of these studies which have attempted to define reliable parameters, including transfusion cut-points to define futility, it has been noted that there are no “consensus guidelines to direct utilization of this resource-demanding intervention and thereby triage appropriate allocation of blood product”. Because of the heterogeneity of the definition of blood products and of the parameters which can reliably predict futility, machine learning (ML) has been proposed as a tool to predict futility by using algorithms and statistical models from patterns in the variations in the data of these patients.

Before one considers the applicability of ML to better define futility in bleeding trauma patients, it is necessary to propose the concept of futility based on criteria for patients whose trauma-induced coagulopathy (TIC) requires MT [3].

Because there is a heterogeneity of measurements and cut-points that have been recently proposed as predictors of futility for SBTPs, it will be useful to define a “futility index”. Recently, the concept of the “never say no” mentality for resuscitating trauma patients warrants reconsideration because of the scarcity of blood products. As a result, there has been a flourishing of literature attempting to define futility for an SBTP. This reflects the urgency of refining those measurements that will reliably define futility for a group of patients who will not only survive, but who represent a costly “component of regionalized trauma care” [4,62]. 

During the recent blood shortages brought about by the COVID pandemic, it was necessary to quickly define imperfect metrics which predicted futility for patients requiring MT caused by trauma-induced hemorrhagic shock. As a result, maximum volume thresholds were established based on age. In addition, during this period of blood shortages, the following markers were used to predict futility: age, pre-arrival cardiac arrest, in-hospital cardiac arrest, initial EV pulse rate from 120 bpm to 160 bpm, TBI with midline shift, fixed and dilated pupils, transfusion amount, initial hospital GCS score, and head injury type (blunt or penetrating) [63]. This study has served as a precursor to recent publications which have attempted to define reliable parameters which predict futility. 

### 1.3. Defining FR and a “Futility Index” Based on Criteria for Trauma-Induced Coagulopathy and Massive Transfusion with Laboratory and Clinical Markers

The use of pre-hospital and ED WB during MT has further increased demands on the blood banking community in addressing the administration of blood components to SBTPs [21,22,23,30,36,55,64,65,66,67,68,69,70,71]. This added pressure on blood banks to provide increased number of blood components and WB has led to a renewed attempt to refine indicators of FR in MT for trauma by using simple markers such as age, peak lactic acid levels, and nadir pH [9,30,31,41,56,72]. Since prior research on critically ill patients with cardiac arrest and/or sepsis has demonstrated that bedside markers such as pH and lactic acid levels are strongly associated with increased mortality, recent studies have attempted to apply these easily available indicators of severity of illness to the identification of patients who will not survive severe traumatic hemorrhage. The futility index has recently been proposed for SBTPs receiving MT who are not likely to survive. This futility index is currently under a state of evolution and is described by different authors using different parameters to define futility. However, there is a commonality of markers used to define futility, which focus on measurements defining the depth and duration of shock, such as nadir pH < 7, peak lactic acid level > 10, and >10 U/h of PRBCs in a subset of patients older than 65 years old [56]. This has led to recent reviews of the literature suggesting that a futility index, which accounts for numerous measurements, may aid in the determination of FR [9,41,56,73,74,75,76]. 

Patients with severe trauma-induced hemorrhage have a high incidence of TIC, which refers to aberrant and maladaptive coagulation progression in proportion to the magnitudes of tissue injury and hemorrhagic shock in severe injury [77,78]. The goal of the traumatologist is to correctly identify patients who are “bleeding because they are dying” as opposed to those who are “dying because they are bleeding” and use highly predictive markers to withdraw resuscitation efforts for the former group of patients with “trauma-induced hemostatic failure”, also referred to as “hemorrhagic blood failure” [1,79]. In a similar fashion to predicting those patients with traumatic hemorrhage who will require MT, traumatologists have sought to predict FR with the same markers used to define TIC [30,31,41,77,80,81,82,83]. MT has evolved from the use of clinical gestalt to the realization that gestalt-driven decisions for determining the need for MT do not accurately predict the true need for MT and underestimate those who may benefit from MT [7,10,13,18,32,84,85,86,87]. Despite various attempts, there has been little progress to accurately predict bedside futility [9,30,31,40,41,56,80,81,88]. Common markers of the adequacy of resuscitation such as vital signs, pH, lactic acid levels, base deficit, and fibrinogen levels are not able to predict futility on their own. However, their use in combination with other clinical and laboratory markers has resulted in a refinement of criteria that may more accurately define FR, as demonstrated by the recent STOP criteria [41]. 

Likened to the prognostic tools for mortality in sepsis and cardiac arrest patients, traumatologists have proposed decision-making tools that adequately define unsurvivable trauma-induced hemorrhage [7,10,13,85]. These tools include clinical scores of illness severity, such as the Sequential Organ Failure Assessment (SOFA) score, which are not easily calculated during the chaotic moments of providing MT to bleeding trauma patients. The SOFA score and other similarly calculated clinical algorithms for futility use data that are not immediately available [7,10,13,89]. Therefore, it seems reasonable to extract reliable and readily available bedside predictors of futility in the setting of MT for trauma from the literature, which independently do not have a 100% PPV and specificity, but when combined may predict futility with a 100% PPV and specificity, or at least improve the quality of the gestalt-based decisions by practitioners. Parameters likely to predict futility would be collected during the immediate and delayed stages of resuscitation. Immediately available markers at the bedside include clinical markers (e.g., age, GCS, and vital signs), number and/or volume of blood products given per hour, and point of care (POC) tests such as viscoelastic tests (VETs). VETs are performed at the bedside, with WB assays measuring the “lifespan of a clot” from initiation, amplification, propagation, and termination via fibrinolysis, which allows for the early identification of deficiencies of coagulation factors, the fibrinogen concentration, fibrin/platelet contraction and clot strength, and finally fibrinolytic phenotype. An example of this technology is noted below [90,91]. The POC tests are listed as immediate since for both thromboelastography (TEG) and rotational thromboelastometry (ROTEM), early descriptions of hemostatic integrity can be seen in the tracing at the so-called “A5” and “A10” parameters, which describe the overall clot strength 5 and 10 min after the initiation of coagulation, respectively. These A5 and A10 values are available within the first 30 min of performance of the VETs. 

The delayed markers and tests are not immediately available at the bedside, such as common coagulation tests (CCTs), partial thromboplastin time (PTT), prothrombin time (PT), international normalized ratio (INR), hematocrit/hemoglobin, platelet count, fibrinogen level, ionized calcium, and laboratory non-bedside pH and lactate tests [77,78,92,93,94,95]. A combination of these immediate and delayed parameters could be applied sequentially during “futility time-outs” (FTOs), “transfusion time outs” (TTOs), or “resuscitation time-outs” (RTOs) as a foundation for determining futility of continuing MT. These “time-outs” will enable traumatologists to reassess the likelihood of survival based on cut-points of blood products given in conjunction with other markers that may predict the likelihood of a favorable physiologic response to resuscitation [9,30,31,32,40,41,56].

Therefore, a systematic review summarizing the scant literature searching for predictable bedside markers has been prompted by the dire shortage of blood components, which is placing an unprecedented strain on blood banks to meet the demands of all who require a transfusion of blood products. Before such a review is undertaken, a description of the markers which have been traditionally used to define futility for severely bleeding patients is in order.

### 1.4. Clinical and Laboratory Measurements for Predicting FR

The same markers that guide the initiation of MT also form the foundation for traumatologists to predict FR. To date, there is no prevailing consensus on a group of markers capable of reliably predicting FR at the bedside during resuscitation for traumatic hemorrhage [9,30,31,32,40,41,51,56,81,88]. There are four main categories of markers that have been studied to predict FR, which are grouped into two categories: immediate (immediate is defined as the first 30–60 min after arrival in the ED) and repeated (repeated is defined as testing after the first 1–4 h post admission to the ED).

**Figure 1:**
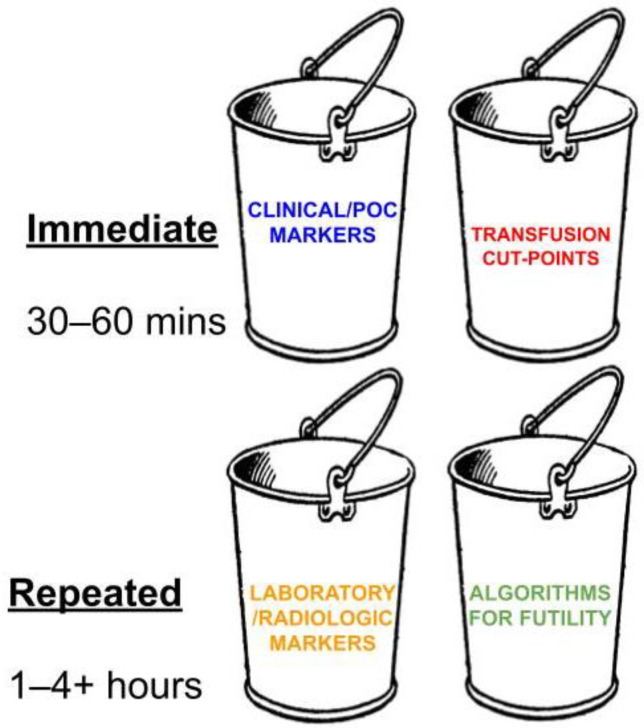
The four categories of investigated futility markers, shown as buckets, used in attempts to define futility in SBTPs. The top two buckets, immediate bedside/clinical markers (e.g., vital signs, GCS, pupil reactivity) with POC testing (pH, lactate, HCO_3_, base deficit, VETs) and transfusion cut-points (units of packed red cells), are markers that are immediately available at the bedside. The bottom two buckets, comprising laboratory markers and algorithms, are repeated markers which take time to obtain results or calculate. Not all trauma centers have immediate availability of bedside testing for pH, lactate, base deficit, bicarbonate, and other POC tests. For those trauma centers with such POC testing, certain parameters of futility such as pH, lactate levels, base deficit, bicarbonate levels, and other parameters may be acquired in the first 30 min after arrival to the ED. See Table 1 for the individual markers and their evidence in each category.

(1) Immediate clinical/bedside POC and radiologic markers (bedside POC testing, VET POC testing, and laboratory results);
(2) Transfusion cut-points;
(3) Repeated laboratory and radiologic markers;
(4) Algorithms for futility (Figure 1) [88].

The definition of “immediate” is not standardized in the trauma literature, yet with the introduction of POC testing including VETs to guide resuscitation, the time frame for defining immediate has been proposed to be within the first 30–60 min following arrival in the ED [88]. This broad strategy of defining immediate for the purposes of developing parameters for determining futility derives from what has been called the “Platinum 10 Minutes” and the “Golden Hour” of trauma [96]. 

Recently, there has been an attempt to define each patient’s unique hemostatic phenotype to personalize resuscitation. This approach applies precision-based medicine (PBM) techniques, such as VETs, to assess the hemostatic competence of bleeding trauma patients. VETs are widely applied not only for predicting those patients who will require MT, but also to guide individualized ratios of blood components during MT [77,78,97,98,99]. An outgrowth of this novel concept of PBM has been the use of VETs as adjuncts for defining FR in SBTP. Traditionally, VETs have been considered a bedside marker for defining the hemostatic phenotype of bleeding and clotting patients and, therefore, a useful test for defining the hemostatic integrity of a patient and their likelihood of survival in the immediate and delayed periods of resuscitation. There is nascent literature regarding VETs as predictors of FR in the surgical and trauma populations [40,80,81].

The literature reveals a wide heterogeneity of markers used to identify futility, further complicating comparisons. Table 1 describes the clinical and laboratory markers that have been used to describe FR. The clinical markers include age, sex, race, mechanism of injury, severity of head injury, traumatic cardiac arrest with or without (ROSC), GCS score, heart rate, SBP, respiratory rate, end-tidal carbon dioxide (ETCO_2_), oxygen saturation/carrying capacity, temperature, signs of life upon arrival, shock index, presence of aortic cross clamp, thoracotomy, endotracheal intubation, absence of cardiac activity with Pericardial Focused Assessment with Sonography in Trauma (P-FAST), resuscitative endovascular balloon occlusion of the aorta (REBOA), and inotrope use. The laboratory markers include base deficit/excess, pH, lactic acid level, hematocrit/hemoglobin level, PT/INR/PTT, platelet count, and fibrinogen count. The transfusion markers include volume of total blood component used, including WB, PRBCs, FFP, PLTs, with or without liquid plasma, cryoprecipitate, factor concentrate, volume of WB transfusion, and volume of crystalloid and colloid used during resuscitation (See Table 1).

The STOP criteria use prehospital parameters in an attempt to define futility more consistently. However, in the prehospital environment for patients with traumatic cardiac arrest, the inconsistencies of guidelines concerning the appropriate management of these patients have resulted in medical directors’ and EMS providers’ uncertainty pertaining to the definition of futility during the early moments of trauma resuscitation for patients [41,100,101]. In an attempt to refine the clinical and laboratory markers, the use of blood components per hour to define a cut-point beyond which resuscitation is no longer beneficial has recently been proposed as the foundation of a futility index, which uses supporting clinical and laboratory markers to confirm FR [9,30,31,32,40,41].

Having considered the parameters which have been used to define futility in bleeding trauma patients receiving MT, a systematic review can be described. 

## 2. Methods

**Figure 2:**
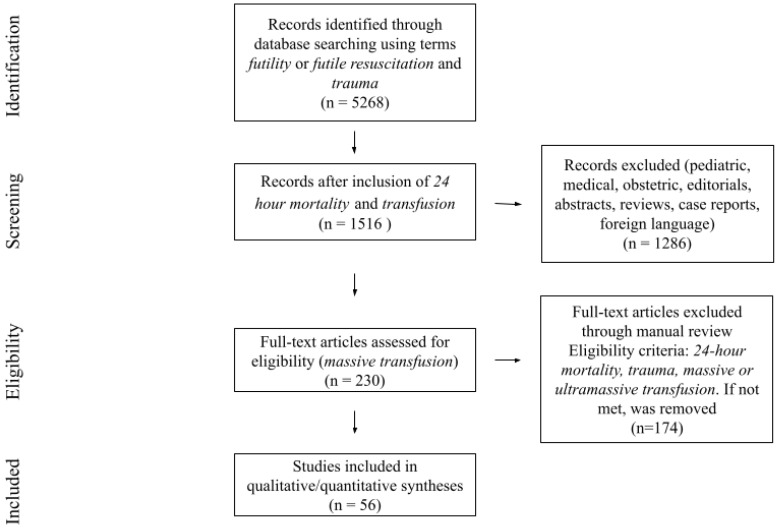
The Preferred Reporting Items for Systematic Review and Meta-Analysis (PRISMA) methodology identifying relevant literature on predicting futility for SBTPs.

The Preferred Reporting Items for Systematic Review and Meta-Analysis (PRISMA) methodology was used to identify relevant literature on predicting futility for SBTPs to construct Table 1 [102]. The databases PubMed, Ovid, Embase, and Cochrane Analysis were searched using the All Fields search and the terms futile or futile resuscitation and trauma, which was refined by the addition of 24 h mortality and transfusion. We applied the exclusion criteria of titular search terms pediatric/paediatric, medical, obstetrics, as well as editorials, abstracts, reviews, case reports, and papers published in foreign languages. In addition, papers containing non-trauma-related keywords were also excluded. Eligibility for analysis of the 230 remaining full text papers was further refined by manually examining the papers for the inclusion criteria of futility or 24 h mortality, trauma, and massive/ultramassive transfusion. Only papers where all subjects were trauma patients who received massive/ultramassive transfusion and analyzed potential markers for mortality within 24 h were selected. As a result, 55 papers fulfilled these criteria. Throughout the entire search, the authors sought papers that were either qualitative or quantitative, and prospective or retrospective cohort studies. There were no randomized controlled trials, meta-analyses, or systematic reviews among these papers. Each title and abstract were screened independently by six members of the working group, with irrelevant studies being discarded. Then, the full texts of the remaining articles were independently screened by four independent working group members and later confirmed by other members of the Futile Indicators for Stopping Transfusion in Trauma (FISTT) collaborative group. The selected studies were included for the final data extraction and analysis. All disagreements between the reviewers were adjudicated by discussion and consensus among the individuals. When a consensus was not reached, an independent and additional reviewer was involved as an arbitrator (Figure 2).

## 3. Results

Table 1 is a summary of the literature regarding bedside laboratory, clinical, and transfusion markers as statistically significant vs. statistically not significant predictors of futility during massive and/or ultramassive transfusion for traumatic hemorrhagic shock in the civilian population. The data for clinical, laboratory, transfusion markers and from algorithms were analyzed.

**Table 1:** Clinical markers of futility (death within approximately 24 h of admission) in adults (age 18+), categorized by statistical significance (according to either univariate or multivariate analysis).

Category of Common Markers	Proposed Marker of Futility	Statistically Significant	Not Statistically Significant
**Clinical markers**	**Age**	Aichholz [103], Barbosa [104], Farrell [81], Dorken-Gallastegi [105], Hamidi [106], Huber-Wagner [107], Mitra [108], Mitra [109], Moore [110], Morris [111], Mostafa [112], Sharpe [6], Torres [113], Velmahos [114], L’Huillier [46]	Barbosa [115], Cinat [116], Criddle [117], Cripps [118], Hanna [55], Heidary [119], Katirai [120], Loudon [31], Matthay [47], Meyer [38], Mitra [121], Muldowney [85], Murray [122], Shea [123], Van Gent [41], Vaslef [59], Yang [124], Yu [53]
	**Sex**	Cinat [116], Dorken-Gallastegi [105], Hamidi [106], Sharpe [6], Torres [113], Yu [53]	Barbosa [104], Barbosa [115], Criddle [117], Cripps [118], Farrell [81], Hanna [55], Heidary [119], Huber-Wagner [107], Katirai [120], Loudon [31], Matthay [47], Meyer [38], Mitra [121], Mitra [109], Moore [110], Mostafa [112], Muldowney [85], Van Gent [41], Vaslef [59], Velmahos [114], Yang [124]
	**Race**		Meyer [38], Mostafa [112], Van Gent [41], Velmahos [114]
	**Mechanism of injury**	Dorken-Gallastegi [105], Hanna [55], Heidary [119], Mitra [108], Meyer [38], Muldowney [85], Sharpe [6], Torres [113]	Barbosa [104], Barbosa [115], Cinat [116], Cripps [118], Criddle [117], Farrell [81], Hamidi [106], Huber-Wagner [107], Loudon [31], Matthay [47], Moore [110], Mostafa [112], Vaslef [59], Van Gent [41], Velmahos [114], Yu [53]
	**Severity of head injury**	Barbosa [104], Borgman [125], Dorken-Gallastegi [105], Matthay [47], Mitra [121], Mitra [108], Muldowney [85]	Cripps [118], Hamidi [106]
	**Traumatic arrest followed by ROSC**	Aichholz [103]	Van Gent [41]
	**GCS score**	Barbosa [115], Cripps [118], Dorken-Gallastegi [105], Hamidi [106], Hanna [55], Huber-Wagner [107], Loudon [31], Matthay [47], Mitra [108], Mitra [109], Morris [111], Mostafa [112], Shea [123], Torres [113], Van Gent [41], Vaslef [59], Velmahos [114], Yu [53], Schneider [51], L’Huillier [46]	Barbosa [104]
	**Heart rate**	Dorken-Gallastegi [105], Hamidi [106], Matthay [47], Mitra [108], Torres [113]	Barbosa [104], Barbosa [115], Borgman [125], Cripps [118], Deep [126] Loudon [31], Mitra [121], Mitra [109], Moore [110], Morris [111], Velmahos [114], Yu [53]
	**Systolic blood pressure**	Borgman [125], Cosgriff [127], Cripps [118], Deep [126], Dorken-Gallastegi [105], Hamidi [106], Hanna [55], Heidary [119], Huber-Wagner [107], Loudon [31], Mitra [108], Mitra [109], Moore [110], Sharpe [6], Torres [113], Van Gent [41], Tzeng [89]	Barbosa [104], Barbosa [115], Cinat [116], Matthay [47], Mitra [121], Morris [111], Velmahos [114], Yu [53]
	**Respiratory rate**		Barbosa [115], Dorken-Gallastegi [105], Mitra [121], Mitra [109], Mitra [108]
	**ETCO_2_**	Dudaryk [128], Bryant [129], Stone [20]	
	**Pulse oximetry and tissue oxygenation**	Dorken-Gallastegi [105], Moore [110]	Velmahos [114]
	**Temperature**	Borgman [125], Cinat [116], Cosgriff [127], Mitra [121], Mitra [108], Velmahos [114]	Barbosa [104], Barbosa [115], Cripps [118], Hamidi [106], Mitra [109], Moore [110]
	**Shock index**	Arslan [15], Hosseinpour [130]	Aichholz [103], Loudon [31], Matthay [47]
	**Aortic cross clamp**	Matthay [47], Velmahos [114]	
	**Thoracotomy**	Dorken-Gallastegi [105], Loudon [31], Matthay [47], Muldowney [85], Yu [53]	Huber-Wagner [107], Van Gent [41], Velmahos [114]
	**Endotracheal intubation**		Dorken-Gallastegi [105], Hamidi [106], Huber-Wagner [107]
	**Presence of free fluid from FAST**		Mitra 2007 [121], Velmahos [114]
	**Absence of cardiac activity with P-FAST**	Israr [131]	
	**Presence of REBOA balloon expansion**	Anand [132]	Matthay [47]
	**Use of inotropes**	Velmahos [114]	
	**Urgent surgery (laparotomy)**	Mitra [108]	Matthay [47], Mitra [121], Yu [53]
	**Urine output**	Velmahos [114]	
**Laboratory markers**	**Base deficit/excess**	Barbosa [115], Borgman [125], Cinat [116], Criddle [117], Cripps [118], Heidary [119], Huber-Wagner [107], Matthay [47], Shea [123], Van Gent [41], Vaslef [59]	Barbosa [104], Farrell [81], Moore [110]
	**pH/acidosis**	Barbosa [115], Cinat [116], Cosgriff [127], Cripps [118], Katirai [120], Mitra [121], Mitra [108], Moore [110], Muldowney [85], Vaslef [59], Velmahos [114]	Barbosa [104], Farrell [81], Van Gent [41]
	**Lactic acid level**	Arslan [15], Matthay [47], Mitra [108], Sharpe [6], Shea [123], Van Gent [41]	Barbosa [104], Mitra [109], Vaslef [59]
	**Hemoglobin/hematocrit**	Barbosa [115], Borgman [125], Cripps [118], Matthay [47], Mitra [108], Moore [110]	Barbosa [104], Cinat [116], Huber-Wagner [107], Mitra [121], Mitra [109], Muldowney [85], Heidary [119], Shea [123], Vaslef [59], Velmahos [114]
	**PT/INR/PTT**	Barbosa [115], Borgman [125], Cripps [118], Heidary [119], Huber-Wagner [107], Matthay [47], Mitra [121], Mitra [108], Moore [110], Muldowney [85], Shea [123]	Barbosa [104], Mitra [109], Van Gent [41]
	**Platelet count**	Aichholz [103], Barbosa [115], Borgman [125], Cinat [116], Cripps [118], Heidary [119], Matthay [47], Mitra [121], Mitra [108], Muldowney [85], Shea [123]	Barbosa [104], Moore [110]
	**Fibrinogen count**	Cinat [116], Mitra [121], Mitra [108], Muldowney [85]	Barbosa [104], Barbosa [115]
	**Bicarbonate**	Mitra [121], Mitra [108]	Mitra [109]
	**Serum calcium**	Velmahos [114]	
	**Serum potassium**	Velmahos [114]	
	**TEG/ROTEM**	Chapman [80], Van Gent [41], Farrell [81], Shea [123]	Matthay [47]
**Transfusion markers**	**Number of blood components transfused (WB, PRBCs, FFP, platelets, with or without liquid plasma or cryoprecipitate analyzed together)**	Mitra [121], Quintana [32]	Cinat [116], Clements [30], Muldowney [85], Van Gent [41], Vaslef [59], Velmahos [114]
	**Number of PRBC units transfused**	Arslan [15], Barbosa [115], Cripps [118], Deep [126], Dorken-Gallastegi [105], Hamidi [106], Huber-Wagner [107], Liu [133], Loudon [31], Mitra [108], Morris [111], Mostafa [112], Quintana [32], Vaslef [59], Yang [124]	Criddle [117], Como [134], Farrell [81], Matthay [47], Mitra [109], Yu [53]
	**Number of FFP units transfused**	Cripps [118], Hamidi [106], Loudon [31], Mitra [108], Morris [111]	Cinat [116], Criddle [117], Deep [126], Farrell [81], Matthay [47], Mitra [109], Vaslef [59], Yang [124]
	**Number of platelet units transfused**	Loudon [31], Morris [111], Quintana [32]	Cinat [116], Criddle [117], Cripps [118], Farrell [81], Vaslef [59], Velmahos [114]
	**Number of cryoprecipitate units transfused**	Farrell [81], Matthay [47]	Cinat [116], Criddle [117], Vaslef [59]
	**Whole blood transfusion**	Clements [30]	Cinat [116], Muldowney [85]
	**Number of crystalloid or colloid units transfused**	Cripps [118], Deep [126], Heidary [119]	
	**Factor VII use**		Borgman [125], Matthay [47]
	**CAT+**	Hu [72], Meyer [38], Stone [20]	
	**RI4+**	Hu [72], Meyer [38], Rahbar [135]	
**Algorithms**	**STOP, TIC, NBTC TTMTP**	Van Gent [41], Eitel [136], Doughty [7]	

Abbreviations: CAT, critical administration threshold; ETCO_2_, end-tidal carbon dioxide; FAST, focused assessment with sonography in trauma; FFP, fresh frozen plasma; GCS, Glasgow Coma Scale; INR, international normalized ratio; LY30, lysis at 30 min; NBTC TTMTP, National Blood Transfusion Committee Triage Tool for Massively Transfused Patients; P-FAST, pericardial focused assessment with sonography in trauma; PRBCs, packed red blood cells; PT, prothrombin time; PTT, partial thromboplastin time; REBOA, resuscitative endovascular balloon occlusion of the aorta; RI, resuscitation intensity; ROSC, return of spontaneous circulation; STOP, Suspension of Transfusion and Other Procedures; TEG, thromboelastography; TIC, trauma-induced coagulopathy; WB, whole blood.

As can be seen from Table 1, there are numerous markers that have been explored in attempts to define specific cut-points for determining which traumatic hemorrhage patients resuscitation should be withdrawn from. The literature has sought to describe specific cut-points that can define FR for bleeding trauma patients with a 100% PPV and specificity [18,30,31,32,40,41,80]. However, the recent literature has also reaffirmed trends that have been noted throughout the history of defining FR in the setting of trauma resuscitation and focused on blood component use per hour as anchor cut-points to be used at the bedside during resuscitation [30,31,32,41]. Most research has focused on determining a transfusion threshold, based on the number of PRBC units transfused, above which, mortality increases significantly [45,107,114,121,133,137]. However, the inclusion of total blood components with or without liquid or lyophilized plasma and/or cryoprecipitate as well as the use of WB have complicated comparisons between studies that attempt to define FR [30,31,36,41,72]. The cause of the complexity in comparing past studies regarding the number of blood components per hour resides in not only the use of different units of blood components per hour to define FR, but also in the different definitions of what constitutes MT [36,72,138]. As has been noted, while MT increases survival for those patients in need of blood components during damage-control resuscitation, there is no accurate, commonly accepted cut-point to determine when MT is futile [30,31,36,41,46,51,72].

## 4. Discussion

Table 1 defines the clinical and laboratory markers that have appeared in the literature on predicting futility. Also noted in Table 1 are transfusion cut-points per unit of time, as well as algorithms which have been used to predict futility in SBTPs. While it is much more straightforward to rely on standard clinical and laboratory markers to define futility, the use of transfusion cut-points has remained an area of significant controversy because of a lack of agreement regarding what constitutes a unit of blood, and the ability of these transfusion cut-points to accurately predict death in SBTPs. This has led to a recent attempt to define (in more granular detail) a combination of clinical and laboratory markers with transfusion cut-points using algorithms to predict futility.

### 4.1. Heterogeneity of the Definition of Unit of Blood Per Hour as a Predictor of FR 

The same markers used to guide the initiation and definition of MT also form the foundation that permits a traumatologist to predict FR. MT has historically been defined as the provision of ≥10 U of PRBCs in 24 h. This number was chosen because it represents the transfusion of an average adult’s entire blood volume. However, this definition is arbitrary, prone to survivor bias, and mainly useful for retrospective analysis [53]. There have been many definitions regarding blood transfusion that are dependent on the term MT and UMT, for example, ultramassive transfusion (UMT) was defined historically as >20 U of PRBCs in 24 h [30,47,105]. In addition, other descriptions of what constitutes MTP, such as the critical administration threshold (CAT+), the resuscitation intensity (RI), as well as the use of different units of PRBCs/hour and a special equation taking into consideration the use of WB, have resulted in a significant heterogeneity in the definition of MT [3,38,86,135,139,140,141,142,143]. Due to the non-standardized methods of defining those who need MT and those who will have FR, none of these definitions based on PRBC or blood components have allowed for an accurate anticipation of the patients who would certainly die. In addition, WB use in urban American trauma centers has increased [21,22,23,24,30,36,55,64,65,66,67,69,70,71,72,95,96]. The result of the prehospital and hospital trauma protocols which now use WB complicates the creation of a transfusion cut-point marker, which not only defines those who will need MT, but also identifies those SBTPs for whom resuscitation would be futile. The incorporation of WB in algorithms predicting MT and futility has led to a score which requires the tallying of WB with PRBCs to predict early mortality at the bedside.

Many level I and level II trauma centers have noted the difficulty of reliably counting blood products during the chaotic administration of different blood components in the short time span following severe trauma. Multiple methods of quantifying real-time blood product administration during MT have included electronic medical record options such as electronic counters or the stacking of empty unit bags on the floor [144]. 

The use of a transfusion cut-point is an easy marker to follow with a whiteboard attached to the rapid infuser, which can accompany the patient from the ED through the radiology suite and subsequently to the operating room and surgical intensive care unit. Whether PRBC units/hour or total blood components/hour is used, the accuracy of FR prediction can be augmented by accompanying and immediately available clinical markers. A problem with many of these clinical markers is that some, such as GCS score, can be affected by the paralysis and sedation of intubated patients, while others cannot be calculated immediately, such as the SOFA score, injury severity score (ISS), and abbreviated injury severity score (AIS) [10,89,104]. Moreover, the acquisition of metabolic and coagulation data and the subsequent entry of these data into algorithms often takes time, which renders them not useful for bedside resuscitation [89,145]. The algorithms are not necessarily meant for bedside use, although they use delayed markers which can be accessed within an hour of the laboratory draw.

### 4.2. Bedside Algorithms Specifically for Defining FR in SBTPs

The proposed algorithms for predicting FR use a combination of clinical data and metabolic, coagulation, and viscoelastic markers to estimate the likelihood of futility. However, many of these algorithms require the calculation of entities such as the AIS, Trauma Revised Injury Severity Score (TRISS), ISS, GCS score, and SOFA score, which are either too time-consuming to calculate at the bedside or, in the case of GCS, are affected by neuromuscular paralysis and sedation [104,145]. To date, few bedside prediction tools have been proposed that allow for high-level early prediction of FR in SBTPs such as the Trauma Early Mortality Prediction Tool (TEMPT) which predicts death by 28 days [146]. Table 2 lists three of the most recently published bedside algorithms for predicting FR in bleeding hemorrhagic trauma patients.

Three recent bedside algorithms are the STOP, TIC, and National Blood Transfusion Committee Triage Tool for Massively Transfused Patients (NBTC TTMTP) algorithms. The STOP and TIC criteria can be calculated at the bedside. For geriatric trauma, it is known that age itself is an important determinant of mortality in geriatric trauma [147]. In addition, the so-called “transfusion futility threshold (TFT)”, which is the amount of transfused blood product after which the odds of mortality do not improve with additional transfusion, have not been defined for the geriatric population. It is also known that frailty independently predicted death or discharge to a skilled nursing facility. However, it is acknowledged that frailty does not significantly influence the TFT in geriatric trauma patients [46,148]. As a result, even for geriatric trauma, there is a paucity of accepted guidelines which specifically define futility for this group of highly vulnerable patients. This situation can be compared to the many decades of application of the age-incorporated futility model for thermal injury such as the Baux Score or revised Baux score (rBAUX) [46,149,150,151]. This review does not concern thermal injury and excludes these findings from the present literature search since we are concerned with defining parameters that identify futility for patients in hemorrhagic shock. The Geriatric Trauma Outcome Score (GTOS) was an attempt to create an algorithm which incorporates age and ISS into a model to calculate the likelihood of death. This score is calculated using an equation which utilizes the age, ISS, and number 22 if the patient has received PRBCs: Age + (ISS × 2.5) + 22 (if given PRBCs) [2,151,152,153,154]. 

However, it is important to note that the ISS is not calculated within the first hours of resuscitation at the bedside, and the ISS and GTOS are calculated 24 h after admission. Rather, the ISS score is used retrospectively and is not appropriate for bedside use [155].

The STOP criteria (Table 3), which can be predicted at the bedside, uses predictable cut-points, combining indicators of shock, such as degree of fibrinolysis and serum lactate level, that together forecast futility of resuscitation with a 100% PPV. FTOs at certain points of MT have been proposed based on STOP criteria [41]. TEG LY30 >30% in conjunction with lactate values as low as 10 mmol/L may predict 100% mortality rates. Even in patients suffering traumatic arrest followed by ROSC, lower LY30 and lactate values were also able to forecast 100% mortality [41]. The STOP algorithm is a bedside algorithm for adults, which was specifically designed to assist in addressing the need for an early identification of futility for severely bleeding adult trauma patients. 

A recently developed bedside marker for severe TIC is called the Trans-Agency Consortium for Trauma-Induced Coagulopathy (TACTIC) score. The TACTIC score is a quantitative scoring system for TIC based on a Likert scale that applies laboratory and TEG values of patients who received MT. Higher TIC scores correlate with imminent mortality, whereby coagulopathy is defined as the clinical inability to develop a thrombus, as manifested by bleeding [83,136,156]. Although this algorithm is not specifically directed to predicting FR, the emphasis is on using rapidly available bedside markers which can identify those patients at greatest risk for immediate death due to hemostatic failure.

Higher immediate post-trauma TIC scores correlate with derangements of conventional coagulation tests and VETs (e.g., the degree of hyperfibrinolysis), as well as the need for MT. Together, these findings portend significant increases in short-term mortality. The TIC score was able to distinguish mechanical bleeding from coagulopathic bleeding because of TIC. In the TIC score, the definition of MT only counts PRBC units, which are guided by clinically observable bleeding rather than laboratory indications of coagulopathy, as other blood products are given based on goal-directed VETs. This finding supports previous studies which indicated that the addition of PLTs and cryoprecipitate did not increase the correlation with laboratory results, and even created intervention bias. Finally, a stratified analysis of traumatic brain injury (TBI) demonstrated that the TIC score performed equally well in the non-TBI and TBI groups [39,77,136,157,158].

The TIC algorithm addresses the confounding factor of TBI. Typically, patients who suffer severe traumatic hemorrhage with accompanying unsurvivable head injury are not candidates for resuscitation based on historically low rates of survival, yet even in patients with severe TBI associated with cardiac arrest, death is not inevitable. For example, a low GCS score and absence of bilateral pupillary reactivity have been shown to be excellent markers of FR in this group of patients. Therefore, for severely hemorrhaging patients whose clinical course is complicated by severe TBI, the use of the GCS and nonreactive pupil activity would help guide the cessation of resuscitation efforts [89,159,160,161,162,163]. 

These three recently published algorithms represent the latest attempts to apply readily available bedside tests with high correlations with mortality for those patients with a traumatic injury who require MT. Recent applications of machine learning have suggested that a cumulative score can predict with certainty the futility of continued resuscitation for patients receiving MT for trauma-related hemorrhage [10]. An example of a machine learning tool is the so-called “futility index”, which uses the combination of a nadir pH of <7, peak lactate level > 10.0 mmol/L, and age > 65 years [56]. However, previous studies have demonstrated that a significant number of patients older than 65 years with severe acidosis recovered [76,114,164,165]. The STOP criteria approach is the most recent non-machine learning algorithm, which uses multiple combinations of clinical and laboratory markers with a 100% PPV for FR [41]. In addition, the TACTIC criteria can further refine the identification of those patients with severe coagulopathy who may not respond to resuscitation due to hemostatic failure in the form of early TIC. Both the STOP and TIC algorithms rely on VETs. In the future, as the refinement of easily accessible and reproducible criteria to predict FR evolve, a machine learning algorithm with artificial intelligence possibilities could be developed for FR, similar to the machine learning algorithms for the prediction of those patients that will require MT [166,167].

Complicated machine learning and statistical analyses have been proposed to define quality metrics for model validations [10]. However, these decision tree analyses remain an academic pursuit and, to date, do not assist traumatologists during the hectic moments of resuscitation. A more straightforward approach would be to use recently proposed transfusion cut-points of “heroic” resuscitation to trigger FTOs, which are then supplemented by clinical and laboratory markers. For example, as noted by Loudon et al. [31], 16 U of PRBCs per 4 h was deemed “heroic” resuscitation (where resuscitation is possible, yet unlikely) and 36 U per 4 h was FR (where resuscitation is not possible). These cut-points would then be reviewed using the STOP criteria (Table 3) to determine the likelihood of continued success. For simplicity and blood product conservation, an earlier cut-point such as 5 U of PRBCs per hour to suggest heroic and 10 U of PRBCs per hour to define futile could serve as hourly cut-points, which would prompt FTOs. 

A hypothetical example of a projected score calculator would be a readily available database from the bedside with the following markers: number of PRBC units per hour, patient age, SBP, temperature, ETCO_2_, GCS score, TEG LY30, and pupillary reactivity as the immediate markers. Delayed markers may include lactate level, ionized calcium level, INR, pH, and base deficit. With a uniform standard of counting only PRBC units and applying reproducible and easily accessible clinical and delayed markers, future prospective studies may enable the calculation of a futility index which could be validated by randomized trials. Armed with such a reliable score calculator, traumatologists could suggest FTOs more often during resuscitation [10]. 

This immediate period of parameter analysis would then be followed with a calculation of clinical, laboratory, and radiologic scores as a second delayed tier, which may then be quickly scored together to determine futility during FTOs [40,41].

The addition of PLTs and cryoprecipitate in a prediction tool has not improved the correlation with outcome data and introduced intervention bias. Therefore, the previous literature has foreshadowed the likelihood that counting PRBC units alone is an important cut-point marker for FR and will outperform the use of partial or total blood components as markers for FR [39,136].

### 4.3. Defining Early Futility Markers during FTOs: Bedside Transfusion Cut-Points, Serial VETs, and Resolving Heterogenous Definitions of Transfusion Cut-Points

We have seen that the recent adoption of blood component units/hour as opposed to solely PRBC units per hour as a cut-point confounds the ability to rely on past literature attempting to define FR [30,31,40]. In addition, the recent adoption of WB in the prehospital, ED, and operating theater settings has complicated the ability of traumatologists to predict FR during FTOs because of the impossibility of immediately estimating the number of total blood products by looking at a whiteboard [36,144]. Simply put, a trauma surgeon knows when “the red stuff goes in” but cannot easily calculate from a whiteboard the timing or volume of the “yellow and white stuff” (i.e., plasma, PLTs, and fibrinogen concentrates) at the same time. Since most blood component therapy during MT approximates a 1:1:1 ratio, it is unnecessary to calculate the amount of FFP, PLTs, with or without liquid or lyophilized plasma, and/or cryoprecipitate [36]. Future studies, hopefully, will resolve this conundrum in favor of the standard transfusion cut-point which has most commonly been PRBC units/hour [30].

A review of the recent literature revealed the differences between papers that report blood component units/hour versus PRBC units/hour. An example can be found in the work by Loudon et al. [31], which defines 36 U of PRBCs given over 4 h as futile. Yet, within the same year, Clements et al. [30] were able to demonstrate a 50% survival rate in patients who were given 50 U of blood components over 4 h. Within the analysis of the paper by Clements et al. [30] is the fact that the number of PRBC units given in the >50 U of components group was 37 U of PRBCs, directly contradicting Loudon’s FR cut-point of >36 U over 4 h. This contrast noted above is a clear example of the confusion wrought by the failure to adhere to a standard definition of what constitutes FR. This confusion is further related not only to the amount of blood products given per hour but also the types of blood products transfused per hour that are used in order to define FR. That is, there is no standardization for defining the amount of blood products per hour, nor the types of blood products given per hour in order to define FR [30,31,168].

Despite this example of the confusion caused by the inconsistent inclusion of blood components that define FR, the most recent literature adapted definitions and scores that incorporate the total number of units of blood products including WB given per hour. While these studies may be of use for purposes of analyzing data for quality assurance, for the busy traumatologists in need of a definitive cut-point, this discrepancy between the amount of PRBCs and different blood components discourages the formation of uniformly accepted data and guidelines. Further complicating the comparison is that Clements et al. and others have used WB while defining blood components to predict FR [30,36,48]. There has been a dramatic increase in the use of WB in urban prehospital and ED settings for the initial resuscitation of SBTPs, although a survival advantage was noted in patients with an increased probability of death based on prehospital and injury characteristics [21,22,23,30,36,55,64,65,66,67,69,70,71,72].

**Figure 3:**
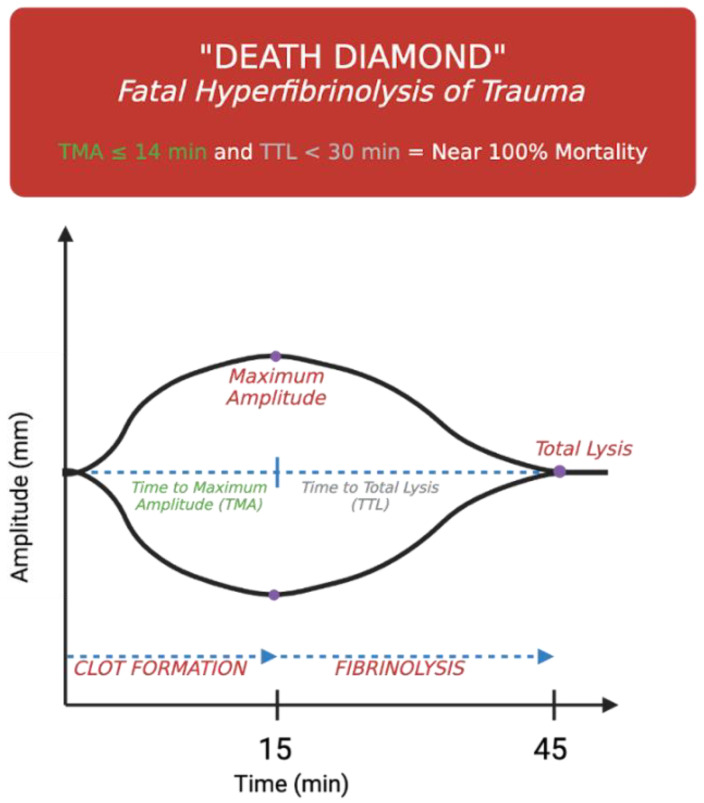
The Death Diamond (DD) is defined as an rTEG tracing with a time to maximum amplitude of ≤14 min and time from maximum amplitude to total lysis of <30 min, which is highly predictive of death. For the TEG 5000, the figure is similar to that of the TEG 6s cartridge system even though the cup and pin system of the TEG 5000 differs from the non-contact LED ultrasound membrane technique of the TEG 6s. In both systems, the TEG patterns are displayed on the screen and the decisions can be made based on a review of the tracing patterns and quantitative parameters [40,80,81,90].

Given the confusion wrought by the varied inclusion of all blood components and WB in the recent literature, the use of a widely accepted and standardized bedside marker of hemostatic integrity and success of resuscitation would be most helpful. VETs have demonstrated great sensitivity and specificity for predicting FR for those patients undergoing resuscitation for trauma-related hemorrhage [40,80,81]. For example, when using VETs, it has been reported that a single rapid TEG (rTEG) parameter indicative of hyperfibrinolysis, described as a short time to maximum amplitude (MA) ≥ 14 min paired with total time to lysis less than 30 min from MA, was universally lethal. This characteristic rTEG pattern is a diamond-shaped tracing (Figure 3), which predicts mortality independent of other clinical or laboratory markers [80]. VETs may serve as the anchor for identifying a patient unlikely to survive as other markers are collected in the trauma bay as resuscitation progresses.

A subsequent follow-up study of serial DDs, called the “Double Death Diamond” (DDD), found a predictive value of 100% mortality. In this study, Farrell et al. described a cohort of trauma patients who with a single DD and a similar 94% mortality as a subset of patients who had 100% mortality if the DD was demonstrated on the subsequent rTEG as well [81]. In this study, the repeated TEG was performed on patients without TBI and occurred within the first four hours of admission. It should also be noted that the authors of the recent STOP criteria commented on increasing the use of LY30 as an independent predictor of mortality. However, the DD tracing specifically was not used in the STOP criteria, likely because of its low incidence [41]. Future studies will need to analyze the timing between VETs and the accuracy of the DDD to predict death as a function of not just the presence of the DDD, but also as a function of the interval between VETs.

The STOP criteria authors also did not use specific transfusion cut-points per hour to predict futility for patients receiving MT for severe trauma-induced hemorrhage. Their exclusion of cut-points may be due to the controversy in the literature regarding the reliability of cut-points to determine futility [30,31,41]. The inclusion of independent markers for the severity of TBI, along with specific transfusion cut-points of blood transfusion per hour, would be most useful in determining the population most likely to be candidates for FR, and that the accuracy of the STOP criteria would be enhanced by the inclusion of TBI severity as well as transfusion cut-points per hour. This proposal is founded on the authors of the STOP criteria, who noted that it is the TBI population who is associated with extracranial trauma that consumes a greater number of blood products [41]. This observation of increased blood consumption of patients with TBI and extracranial hemorrhagic shock could explain the inability of studies to reproduce specific transfusion cut-points as independent markers of futility [7,9,10,13,30,31,32,41]. 

### 4.4. The Proposal for Defining a Futility Check List of All Bleeding Trauma Patients Who Are Candidates for FR with and without Associated TBI

**Figure 4:**
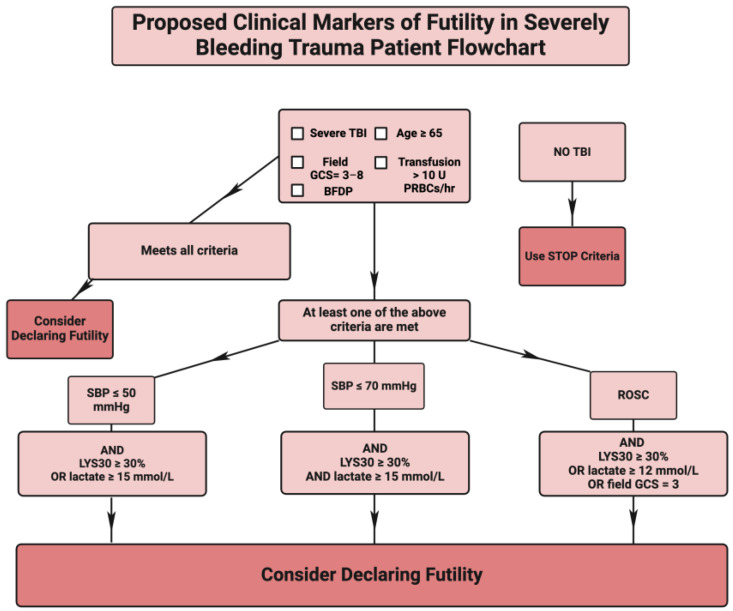
Proposed flowchart to define futility for patients with severe bleeding due to trauma-induced coagulopathy with and without TBI.

Because severe TBI combined with a low GCS score, BFDP, and age > 65 are fairly reliable markers of futility, their addition to transfusion cut-points could form the first step in the process of defining futility during the futile time-out (Figure 4). For those patients who do not meet all the TBI futility criteria (top box of Figure 4), the utilization of the STOP criteria allows for the determination of futility. In addition, patients without a TBI directly depend on the STOP criteria for determining futility. It has been noted by the authors of the STOP criteria that those patients with severe TBI consumed an increased number of blood products with fewer markers of hypoperfusion [41]. A decision tree can be proposed whereby the SBTP is first evaluated for the presence and the severity of TBI. If the patient has severe TBI, a decision regarding futility can be made earlier. For a patient without TBI, the termination of futility can be made by utilizing the STOP criteria. An example of the proposed decision tree is provided in the flowchart in Figure 4. 

The initial step in this flowchart is to determine the presence of severe TBI. Severe TBI combined with a low GCS score, BFDP, and age > 65 are fairly reliable markers of frailty as well as futility. Their addition to transfusion cut-points could form the first step in the process of defining futility during the futility time-out. For those patients who do not meet all the TBI futility criteria (top box of the flowchart), the utilization of the STOP criteria allows for the determination of futility. Assessing patients without TBI will directly depend on the STOP criteria for determining futility [7,9,10,30,31,41,169,170,171].

**Figure 5:**
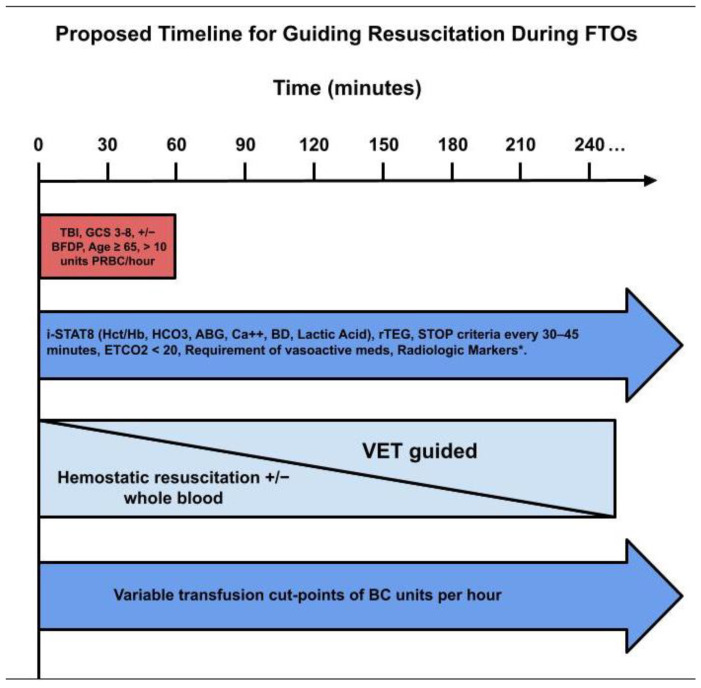
Proposed timeline describing measurements for triggering the consideration of futility during FTOs. No single anomalous measurement defines futility. However, consideration of futility begins if the patient has severe TBI (GCS 3-8), BFDP, is aged >65, and received >10 units of PRBCs/hour. The prognosis of patients with severe TBI as described in this timeline, in the presence of uncontrolled hemorrhage, lowers the threshold of declaring futility and therefore 10 units of PRBCs/hour is proposed as a useful cut-point for determining futility for this group of patients. Futility is also determined by the other clinical and laboratory parameters and variable transfusion cut-points which form the framework for utilizing FTOs to declare futility [163,172,173]. * Radiologic indicators of severity include cerebral edema with or without a midline shift and brain herniation, which is divided into uncal, central trans tentorial, cerebral tonsillar, falcine, upward posterior fossa/cerebellar herniation, and degree of penetrating injury for TBI [163,169,171].

The flowchart in Figure 4 is an attempt to define futility with a 100% PPV and specificity. Following this proposed flowchart for these parameters allows the traumatologist to render a decision regarding futility with much greater accuracy than relying on traditional gestalt [18,41,88]. A timeline for guiding the traumatologist during those early moments of hectic resuscitation, whereby decisions regarding futility are made during FTOs, are proposed in Figure 5, which presents a sequential application of the proposed steps in of Figure 4 flowchart.

Therefore, during the application of the above process, the determination of futility comprises a two-tier bedside prediction tool for patients with and without TBI. Futility is first determined by the severity of TBI and then by markers for the depth and duration of hemorrhagic shock as noted by the STOP criteria [7,9,10,30,31,39,41,81,83,88,131,169,171,174].

## 5. Conclusions and Ethical Considerations

The COVID-19 pandemic, coupled with the increased use of WB and fixed ratios for trauma resuscitation, has heightened the attention to the practical and ethical problems confronting traumatologists when providing blood components to SBTPs. This period in history, recently called a “canary in the coal mine”, provides an impetus for improved stewardship of scarce blood components [13]. The absence of a clinical framework for determining FR leaves room for bias, leading us to propose an immediate/repeated system of immediate clinical/radiologic/bedside POC and repeated laboratory markers (facilitated by PRBC counts and periodic FTOs) which reliably predict the futility of resuscitation for patients in hemorrhagic shock. This immediate/repeated system combines multiple known methods of anticipating FR to optimize the limited blood resources. Moreover, use of these futility indices for trauma resuscitation eases MT decisions by providing high-level guidance driven by reproducible markers that can define FR, saving blood products [10]. Additionally, threshold values for determining FR may need to be altered to account for potential high demands and a low supply [14]. It is evident that other clinical and non-clinical factors impact not only the success of FR prediction, but also how healthcare systems implement this framework in their respective communities. Figure 4 and Figure 5 are examples of a flowchart and a timeline which can be used at the bedside to guide the resuscitation of SBTPs while determining futility during the FTOs.

**Figure 6:**
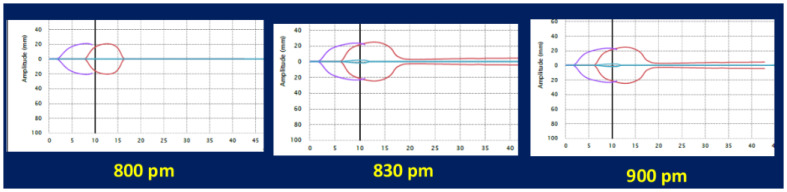
Serial thromboelastography demonstrates the classic early time to maximum amplitude and time to total lysis characteristics of the so-called “Death Diamond” TEG tracing which predicts death in SBTPs in hemorrhagic shock due to TIC (See Figure 3). The red line represents the kaolin TEG, and the purple line represents the rapid TEG. The red kaolin TEGs display the “Death Diamond”.

The three cases described below represent examples whereby literature-based parameters that reliably predict futility can be used in different patient populations dying of trauma-induced hemorrhage. For example, an 80-year-old patient involved in a motor vehicle crash with severe TBI and BFDP who received ten units of PRBCs in one hour to treat bleeding caused by a pelvic fracture and long bone fractures, and is in hemorrhagic shock would not be a candidate for continued resuscitation because of the severity of the head injury combined with the continued hemorrhage due to extracranial injuries. This case is contrasted with a 24-year-old patient with an isolated gunshot wound to the abdomen involving the vena cava, who was also in hemorrhagic shock on admission with initial severe acidosis and hypotension but responded to massive transfusion and damage control resuscitation with a return of blood pressure and acceptable lactate clearance. The transfusion of large quantities of blood into this young patient without TBI is justified and is an example of the value of the flowchart in Figure 4 mentioned above as well as the algorithm in Figure 5 for providing an equitable distribution of blood products to young patients who will be good candidates for survival despite having received large quantities of blood. These two scenarios confirm the importance of including the severity of TBI and age as an initial checkpoint in determining futility and highlight the problems noted in those algorithms that do not include age and the severity of the TBI. On the other hand, in Figure 6, a 27-year-old male who had been shot in the left groin presents to the emergency department in traumatic cardiac arrest. The patient had an immediate resuscitative thoracotomy with cross clamping of the aorta, which resulted in ROSC. Damage control resuscitation was undertaken, and the patient received 107 U of PRBCs in 12 h. Despite repair of his lacerated internal iliac artery and continued resuscitation, the patient expired at 72 h. The three TEG6s below (Figure 6) demonstrate the sequential “Death Diamonds” performed in alignment with the protocol outlined by the STOP criteria and expanded upon in the flowchart of Figure 4 and in the timeline of Figure 5. This case provides an example of the utility of serial TEG6s early in resuscitation which demonstrate the “Death Diamond” as shown in Figure 6. Resuscitation could have been ended earlier in this case had the traumatologist recognized the importance of the failure of this patient to respond to resuscitation. However, rather than relying simply on the presence of the serial “Death Diamond” as a hard and fast determinant of futility, the presence of the “Death Diamond” would prompt a discussion of futility during an FTO which otherwise would not have occurred. This thought process is an example of “metric-assisted gestalt” [163].

Another important element of the development of this framework is its transparency. With respect to FR protocols, this transparency refers to the statistical analyses, surgical success percentage, the team developing the tool, as well as resulting improvements in prognosis and quality of life. Transparency in development can mean inviting blood bankers to participate in the development of a bedside tool for defining futility that considers the current blood bank inventory. The inclusion of the relevant specialties such as surgery, anesthesia, emergency medicine, obstetrics, critical care, and members of the ethics committee in the first moments of creation of FR policy may result in a more successful adoption and adaptation of these markers across many medical disciplines.

Transparency with respect to the patient population is equally paramount. Some patients and families may insist on the continued transfusion of blood, as is their right in a healthcare system built on autonomy. It is therefore crucial that any futility system in place is strengthened by not only its statistical power, but by community outreach and education. Patients and family members who understand the scarcity of blood resources and have the assurance of statistically sound FR guidelines at their institutions can make informed decisions about the cessation of transfusion [175].

The identification of objective markers in the literature will assist in the provision of ethical and transparent guidelines in the use of scarce blood components for SBTPs. Future studies using bedside decision tools that incorporate PRBC/VET, clinical, radiologic, bedside POC, formal laboratory, and algorithmic cut-points will need further validation through meta-analyses and larger studies [7,9,10,13,30,31,40,41,56,85]. Most recently, for example, the Futility of Resuscitation Measure (FoRM) algorithm for futility used the following parameters to define futility: sTBI [GCS ≤ 8], TBI midline shift, craniectomy, nadir in-hospital SBP [≤1 h], prehospital cardiac arrest, 4-h PRBC transfusions, ED resuscitative thoracotomy, REBOA, emergency laparotomy [≤2 h], and early vasopressor requirement [176]. Reliance on such historically derived and consistently reproducible cut-points will be of significant importance in allowing for the ethical and scientifically based distribution of blood components to the patients who require a disproportionate number of blood products.

## Figures and Tables

**Table 2:** Bedside algorithms/clinical scoring systems to predict futile resuscitation. Abbreviations: ED, emergency department; GCS, Glasgow Coma Scale; ISS, injury severity score; LY30, lysis at 30 min; PRBC, packed red blood cell; ROSC, return of spontaneous circulation; SBP, systolic blood pressure; TIC, trauma-induced coagulopathy.

Study	Scoring Criteria
Suspension of Transfusion and Other Procedures (STOP) [41]	The proposed STOP criteria include any one of the combined clinical and lab markers below, each combination having a 100% PPV and specificity for death: Arrival SBP ≤ 50 and LY30 ≥ 30%; Arrival SBP ≤ 50 and lactate ≥ 15; Arrival SBP ≤ 70, lactate ≥15 and LY30 ≥ 30%; Prehospital/ED ROSC and LY30 ≥ 30%; Prehospital/ED ROSC and lactate ≥ 12; Prehospital/ED ROSC and GCS 3.
Trans-Agency Consortium for Trauma-Induced Coagulopathy (TACTIC) TIC Score [136]	Normal hemostasis (negative); Mild coagulopathy, no intervention required except direct pressure or temporary gauze tamponade (equivocal); Coagulopathy refractory to direct pressure, requiring multiple routine hemostasis techniques (e.g., electrocautery, topic hemostatic agents, staples, or suturing) (possible positive); Coagulopathy requiring adjunctive blood component therapy or systemic therapeutics in response to continued bleeding despite the above surgical hemostatic maneuvers (positive); Diffuse persistent bleeding from multiple sites remote from injury, e.g., endotracheal tube, intravenous catheter, chest tubes, etc. (definitive positive); Potential use for future definition of futility.
National Blood Transfusion Committee Triage Tool for Massively Transfused Patients (NBTC TTMTP) [7]	Sequential organ failure assessment (SOFA) score; Total blood components used; Need for ongoing transfusion support; Ability to control bleeding with either surgery or other procedures (e.g., interventional radiology, endoscopy). Patients with a SOFA score > 11, who have a continued need for large amounts of blood components, and where there is no foreseeable ability to control blood loss should be triaged to palliative care.

**Table 3:** Proposed STOP criteria. Data from Van Gent et al. [41]. Adapted with permission from Van Gent [41].

Variable	PPV, %	NPV, %	Sn, %	Sp, %
Arrival SBP ≤ 50 and LY30 ≥ 30%	100	78	33	100
Arrival SBP ≤ 50 and lactate ≥ 15	100	77	31	100
Arrival SBP ≤ 70, LY30 ≥ 30%, and lactate ≥ 15	100	77	30	100
ROSC and LY30 ≥ 30%	100	78	33	100
ROSC and lactate ≥ 12	100	76	29	100
ROSC and GCS 3	100	77	27	100

Abbreviations: GCS, Glasgow Coma Scale; LY30, lysis at 30 min; NPV, negative predictive value; ROSC, return of spontaneous circulation; PPV, positive predictive value; SBP, systolic blood pressure; Sn, sensitivity; Sp, specificity.

## Abbreviations

ABC	Assessment of Blood Consumption
ACS	American College of Surgeons
AIS	Abbreviated Injury Scale
BD	Base Deficit
BFDP	Bilateral Fixed and Dilated Pupils
CAT	Critical Administration Threshold
CCT	Common Coagulation Test
COVID-19	Coronavirus Disease 2019
CT	Computed Axial Tomography
cTBI	Coagulopathy of Traumatic Brain Injury
DD	Death Diamond
DDD	Double Death Diamond
ED	Emergency Department
ERAS	Edinburgh Ruptured Aneurysm Score
ETCO_2_	End-Tidal Carbon Dioxide
FAST	Focused Assessment with Sonography in Trauma
FFP	Fresh Frozen Plasma
FR	Futile Resuscitation
FTO	Futility Time Out
GAS	Glasgow Aneurysm Score
GCS	Glasgow Coma Scale
GTOS	Geriatric Trauma Outcome Score
hMTP	Historic Massive Transfusion Protocol
INR	International Normalized Ratio
ISS	Injury Severity Score
LD50	Median Lethal Dose
LY30	Lysis at 30 min
MA	Maximum Amplitude
MCF	Maximum Clot Firmness
MELD	Model for End-Stage Liver Disease
MeSH	Medical Subject Headings
MT	Massive Transfusion
MTP	Massive Transfusion Protocol
NBTC	National Blood Transfusion Committee
OR	Operating Room
PBM	Precision-Based Medicine
P-FAST	Pericardial Focused Assessment with Sonography in Trauma
PLTs	Platelets
POC	Point of Care
POCUS	Point of Care Ultrasonography
PPV	Positive Predictive Value
PRBCs	Packed Red Blood Cells
PROPPR	Pragmatic Randomized Optimal Plasma and Platelet Ratio Trial
PT	Prothrombin Time
PTT	Partial Thromboplastin Time
rBAUX	Revised Baux Score
RCT	Randomized Controlled Trial
REBOA	Resuscitative Endovascular Balloon Occlusion of the Aorta
RI	Resuscitation Intensity
ROSC	Return of Spontaneous Circulation
ROTEM	Rotational Thromboelastometry
rTEG	Rapid Thromboelastography
RTO	Resuscitation Time-Outs
SBP	Systolic Blood Pressure
SHINE	SHock-INduced Endotheliopathy
SOFA	Sequential Organ Failure Assessment
SOL	Signs of Life
STOP	Suspension of Transfusions and Other Procedures
TACTIC	Trans-Agency Consortium for Trauma-Induced Coagulopathy
TBI	Traumatic Brain Injury
TEG	Thromboelastography
TEG LY 30	Thromboelastography Lysis at 30 Minutes
TEMPT	Trauma Early Mortality Prediction Tool
TFT	Transfusion Futility Threshold
TIC	Trauma-Induced Coagulopathy
tPA	Tissue Plasminogen Activator
TRISS	Trauma Revised Injury Severity Score
TTMTP	Triage Tool for Massively Transfused Patients
TTO	Transfusion Time-Out
TTV	Total Transfusion Volume
TQIP	Trauma Quality Improvement Program
U	Unit
UMT	Ultramassive Transfusion
VET	Viscoelastic Testing
VSS	Vancouver Scoring System
w/o	Without
WB	Whole Blood
